# Borderline personality symptom severity predicts reduced mentalization and affective empathy and altered responses to fairness in the Ultimatum Game

**DOI:** 10.1186/s40479-026-00358-y

**Published:** 2026-07-08

**Authors:** Pascal Louis, Simon Rigoulot, Dominick Gamache, Audrey Bernier, Justine Le Blanc-Brillon, Claudia Savard, Sébastien Hétu

**Affiliations:** 1 Département de psychologie, Université de Montréal, Université de Montréal, 90, avenue Vincent-D’Indy, Montreal, QC H2V 2S9 Canada; 2 Département de psychologie, Université du Québec à Trois-Rivières, 3600, rue Sainte-Marguerite, Local 2008, Pavillon Michel-Sarrazin, Trois-Rivières, QC G9A 5H7 Canada; 3 CogNAC Université du Québec à Trois-Rivières, 3351, boulevard des Forges, C.P. 500, Trois-Rivières, QC G9A 5H7 Canada; 4 BRAMS, Pavillon Marie-Victorin, local A-108 90 avenue Vincent-d’Indy, Outremont, QC H2V 2S9 Canada; 5 CERVO Brain Research Center, CERVO Brain Research Centre, Chemin de la Canardière, 2601, Chemin de la Canardière, Québec, QC G1J 2G3 Canada; 6 Département de psychologie, Faculté des lettres et sciences humaines, Université Sherbrooke, 2500, boulevard de l´Université, Sherbrooke, QC J1K 2R1 Canada; 7 Département des fondements et pratiques en éducation, Pavillon des Sciences de léducation, Université Laval, 2320, rue des Bibliothèques, Quebec, QC G1V 0A6 Canada; 8 CIRCA Research Center, CIRCA – Université de Montréal, Pavillon 3744 Jean-Brillant, Bureau 260-15, Montréal, QC H3C 3J7 Canada; 9 REPERE Research Lab REPERE, Université de Sherbrooke, Pavillon A7, local 025, 2500 Boulevard de l’Université, Sherbrooke, QC J1K 2R1 Canada

**Keywords:** Borderline personality, Community sample, Empathy, Mentalization, Social decision-making, Ultimatum game, Emotion recognition

## Abstract

**Background:**

Borderline personality (BP) symptoms are characterized by emotional instability, impulsivity, and interpersonal difficulties. Deficits in mentalization (ability to attribute mental states to self and others) and affective empathy (congruent and incongruent emotional reactions), may contribute to these social impairments. This study investigated whether, in a community sample, mentalization and affective empathy mediate the relationship between BP symptom severity and altered social decision-making in the Ultimatum Game (UG), where participants were asked to accept/reject fair and unfair monetary offers from Proposers displaying various emotional facial expressions (happy, angry, neutral).

**Methods:**

A community sample of 111 participants completed a measure of BP symptom severity, a dispositional mentalization/affective empathy questionnaire, an emotion recognition task to assess behavioral mentalization, and played the role of Responders in the UG. Relations between BP symptom severity and mentalization and affective empathy measures were tested using hierarchical regressions. Social decision-making was analyzed using multilevel logistic regression models examining the effects of fairness, emotion, and BP symptom severity on acceptance decisions. The potential mediating role of mentalization and affective empathy was evaluated using multilevel mediation analyses.

**Results:**

Higher BP symptom severity was significantly associated with poorer dispositional affective empathy (on both resonance and dissonance scores). Higher BP symptom severity was also associated with lower dispositional mentalization and impaired behavioral mentalization, especially for the recognition of happiness and anger. In the UG, BP symptom severity interacted with fairness of the offer: individuals with higher BP symptom severity were less likely to accept fair offers, whereas acceptance of unfair offers did not differ as a function of symptom severity. BP symptom severity was not associated with differential sensitivity to Proposers’ emotional expressions. Preliminary analyses indicated that mentalization and affective empathy were not associated with behavioral responses in the UG; therefore, mediation analyses were not conducted.

**Conclusions:**

Findings highlight mentalization and affective empathy impairments as important elements of BP symptomatology. However, altered social decision-making associated with BP symptoms appears to be specific to responses to fairness rather than to emotional expressions per se. Further research in clinical populations is needed to clarify the mechanisms underlying these interpersonal decision-making patterns.

**Supplementary Information:**

The online version contains supplementary material available at https://doi.org/10.1186/s40479-026-00358-y.

## Background

Borderline personality (BP) disorder is a complex mental health condition characterized by pervasive patterns of instability in interpersonal relationships, self-image, and emotions, often accompanied by marked impulsivity [4]. Individuals with BP disorder frequently encounter significant social challenges, including heightened loneliness, reduced social networks, and impaired social functioning [21]. Robust evidence suggests that borderline symptoms are best conceptualized dimensionally (e.g., Trull et al., [43], and that dimensional ratings of borderline psychopathology are especially relevant at subthreshold levels, where they may still indicate clinically significant impairment [47]. More broadly, the dimensional perspective on personality disorders—including borderline personality disorder—is reflected in the Alternative Model for Personality Disorders (AMPD) proposed in DSM-5 (e.g., [1, 37, 39, 48]). In this model, borderline personality psychopathology is defined by moderate or greater impairment in personality functioning (i.e., dysfunction in self and interpersonal areas; Criterion A) and specific pathological traits like emotional lability, impulsivity, and separation insecurity (Criterion B). Although sociocognitive factors in BP disorder’s social functioning have been widely studied [2, 5, 22, 29, 34, 36], existing work has largely examined mentalization, affective empathy and social decision-making in isolation and predominantly in clinical samples. The present study addresses this gap by examining BP symptom severity dimensionally in a nonclinical community sample and by jointly assessing mentalization, affective empathy and social decision-making within a single experimental paradigm involving the recognition of emotional facial expressions. Critically, this study is the first to directly test whether mentalization and affective empathy alterations mediate the relationship between BP symptom severity and social decision-making behavior. By adopting an integrative and dimensional approach, the present work provides novel insight into the sociocognitive mechanisms linking BP symptom severity to social behavior.

Potential sociocognitive factors influencing social interactions in BP symptomatology are empathy and mentalization. Although these concepts can be examined within neurobiological or neurocognitive frameworks (e.g., [38]), the present study adopts a psychological and cognitive level of analysis, defining and measuring these concepts as behavioral and dispositional abilities [7, 32, 44]. Empathy is a multifaceted construct encompassing both affective and cognitive components or dimensions. Affective empathy is defined as the sharing or resonating with others’ emotions [7, 44] and is sometimes divided into affective resonance—experiencing emotions that are congruent with the emotional states of others (i.e., sharing their feelings)—and affective dissonance—experiencing emotions that are incongruent with the other person’s emotional state [44]. For instance, perceiving happiness in someone else may evoke a positive emotion in the case of affective resonance, whereas it may elicit a negative emotion in the case of dissonance. Cognitive empathy has traditionally been defined as the ability to identify and understand another person’s mental state or emotion [7, 44]. However, this definition substantially overlaps with the concept of mentalization, defined as the ability to attribute mental states to self and others [32]. Following recent conceptual recommendations [32], we therefore use the term mentalization (about affective states) instead of cognitive empathy throughout the present work.

Affective empathy and mentalization can also be conceptualized in terms of dispositional vs. behavioral. Dispositional affective empathy or mentalization refers to relatively stable traits or tendencies towards others mental states, emotional experiences or expressions and is usually measured with self-reported questionnaires (e.g., “I can easily know when someone is happy or sad” for mentalization; “When someone is happy, I feel good” for affective empathy). Behavioral (state) affective empathy or mentalization is closer to “performance” or accuracy and is usually measured with tasks where participants must identify/recognize or react to others’ mental states, emotional experiences or expressions (e.g., “What is the emotion expressed by this person?” for mentalization; “When looking at this person, what do you feel?” for affective empathy). However, recent meta-analytic and empirical evidence suggest that most dispositional measures of affective empathy and mentalization (cognitive empathy) are only weakly related to their behavioral counterparts [25, 41]. Because these constructs are assessed using different methodologies, scores on dispositional measures should not be interpreted as direct indicators of mentalization or affective empathy abilities, and vice versa. The weak correlations between the two may also indicate that individuals have difficulties accurately evaluating their own mentalization and affective empathic abilities (see interpretation by [25]). Overall, combining dispositional and behavioral assessments may provide a more comprehensive understanding of both individuals’ mentalization and affective empathic tendencies and their actual performance.

Research indicates that individuals with BP disorder may exhibit impairments in both dispositional affective empathy and mentalization which could contribute to their interpersonal difficulties [36]—although results regarding affective empathy are more mixed (e.g., Harari, 2010, [2]) and partly depend on the type of measure used [36]. Less data is available for BP symptom severity relation with dispositional affective empathy and mentalization in the general population. A recent study by Maheux-Caron and colleagues [23] suggests that BP symptoms severity is not associated with dispositional mentalization nor affective empathy when measured as affective resonance. However, it was associated with affective dissonance. When considering behavioral measures, the few studies that have assessed affective empathy in BP disorder or symptom severity suggest reduced resonance [27, 33]. While there has been more work focusing on impairments in behavioral mentalization in BD disorder, results have generally been mixed. The meta-analysis by Daros and colleagues [8] suggests that individuals with a BP disorder diagnosis are less accurate than controls in recognizing neutral and anger facial expressions. However, a recent meta-analysis did not find impairment in mentalization (measured as emotion identification) in individuals with a diagnosis of BP disorder [5]. Furthermore, BP symptom severity in a community sample was mostly not related to behavioral mentalization—it was only associated with better recognition of fear in others [23]. Clarifying how affective empathy and mentalization difficulties manifest in BP symptomatology, particularly in community samples, is therefore essential to understand their potential consequences for social decision-making.

Social decision-making tasks, such as the Ultimatum Game (UG), can provide valuable insights into interpersonal dynamics in nonclinical individuals and those with BP disorder. In the UG, a Proposer offers a division of a money endowment to a Responder, who can either accept or reject the offer [6]. If the Receiver accepts, Receiver and Proposer keep their share of the division; if the Receiver rejects, both receive nothing. Rejections typically occur in response to perceived unfairness, serving as a form of altruistic punishment to enforce social norms [12]. Since this form of norm enforcement is costly (rejections resulting in missing out on some money), it is often conceptualized as a prosocial behavior where individuals choose long-term and altruistic outcomes such as fostering cooperation instead of immediate personal gains. A mini meta-analysis on four studies found no difference in acceptance rate between BP disorder and control groups [10]. However, a study using a modified UG with photographs of proposers with varying emotions reported that individuals with BP disorder are more likely to accept unfair offers compared to healthy controls, suggesting altered social decision-making processes [31]. This pattern has been interpreted as reflecting a reduce tendency to engage in altruistic punishment to enforce social norms or to have difficulties in postponing immediate personal gains over more long-term ones [31]. Furthermore, the proposer’s emotional expressions, such as happiness or anger, can influence acceptance rates in the UG. Indeed, healthy individuals tend to accept offers from happy Proposers more frequently compared to neutral or angry Proposers—this effect being stronger for unfair offers [26]. Interestingly, this effect appears diminished in individuals with BP disorder, indicating a potential reduced sensitivity to social cues or an alteration in how they are processed and used during decision-making [31]. However, it remains unknown whether these disruptions in decision-making during the UG are also associated with BP symptom severity in the general population and whether they are related to affective empathy or mentalization alterations.

It is plausible that alterations in mentalization mediate the relationship between BP symptom severity and social decision-making. Specifically, impaired mentalization could lead to difficulties in accurately interpreting the proposer’s emotional expressions and intentions, resulting in atypical responses during social interactions. This could explain why acceptance patterns in the UG may not systematically vary with the emotion expressed by Proposers (i.e., higher acceptance rate for offers from happy versus angry Proposers). Additionally, affective empathy, encompassing affective resonance and affective dissonance, may further influence these interactions. For instance, poorer affective empathy—on dissonance or resonance—might exacerbate interpersonal conflicts by respectively triggering negative emotional responses or blunting positive emotional responses when observing positive emotions. Feeling contradictory emotions or blunted congruent emotions could lead to a diminished influence of Proposer emotions in the UG usually found in the general population when playing with happy versus angry Proposers [31].

Consequently, this study aims to explore the mediating roles of mentalization and affective empathy in the relationship between BP symptom severity and social decision-making, within the context of the UG involving Proposers displaying various emotional expressions. By employing both a dispositional self-reported assessment—the Affective and Cognitive Measure of Empathy (ACME; [44]), and a behavioral measure—an emotion recognition task—we seek to provide a comprehensive understanding of how mentalization and affective empathy alterations contribute to the social challenges associated with BP interpersonal symptomatology.

### Hypotheses

Building upon existing literature and the objectives of our study, we propose the following hypotheses:

1. Relationship between BP symptom severity, affective empathy and mentalization:

- **H1a**: Higher BP symptom severity will be associated with lower affective resonance—the tendency to share and mirror the emotions of others—as measured by the affective resonance subscale of the ACME.
- **H1b**: Higher BP symptom severity will be associated with lower affective dissonance scores as measured by the ACME affective dissonance subscale (i.e., a greater tendency to experience emotions that are incongruent with those of others, such as feeling happy when others are sad).
- **H1c**: Higher BP symptom severity will be associated with lower mentalization, as measured by both the cognitive subscale of the ACME and an emotion recognition task (correct identifications of happy, angry, and neutral facial expressions). We will also look at the relation between BP symptom severity and patterns of misclassification, as part of exploratory analyses.

2. Relationship between BP symptom severity and social decision-making in the UG:

- **H2a**: Higher BP symptom severity will be associated with an increased acceptance rate of unfair offers in the UG, indicating altered social decision-making strategies.
- **H2b**: Higher BP symptom severity will be associated with reduced sensitivity to the Proposer’s emotional expressions, leading to a diminished influence of Proposer emotions (e.g., happiness or anger) on their acceptance rates of unfair offers.

3. Mediating roles of mentalization, affective empathy in the relation between BP symptom severity and social decision-making in the UG^1^:

- **H3a**: The relationship between BP symptom severity and reduced sensitivity to the proposer’s emotional expressions in the UG will be mediated by alterations in mentalization.
- **H3b**: The relationship between BP symptom severity and reduced sensitivity to the proposer’s emotional expressions in the UG will be mediated by alterations in affective resonance.
- **H3c**: The relationship between BP symptom severity and reduced sensitivity to the proposer’s emotional expressions in the UG will be mediated by alterations in affective dissonance.

These hypotheses aim to elucidate the complex interplay between BP symptom severity, mentalization and two facets of affective empathy (affective resonance, and affective dissonance), and social decision-making processes in contexts involving fairness considerations in the general population.

### Methods

#### Participants

Participants from the United States were recruited via Amazon Mechanical Turk. Initially, 113 participants were retained for analysis; however, one participant was excluded due to technical issues, and one failed our attention check question. The final sample comprised 111 participants (60% men; *M*age = 39.3 years, *SD*age = 11.7). For detailed demographic information, refer to Table 1.

#### Procedures

Participants were directed to the LimeSurvey platform, where they first provided informed consent. They completed various tasks and questionnaires, presented in random order except for the sociodemographic questionnaire, which was always administered last. After disclosing deceptive elements, consent was reaffirmed at the study’s conclusion. The Ethics Committee for Research in Education and Psychology (CEREP) at the University of Montreal approved the project. Participants received a $5 USD compensation for their participation.

#### Materials and measures

##### Sociodemographic questionnaire

Participants provided information on age, sex assigned at birth (Male, Female, Other/Do not wish to answer), gender identity (Man, Woman, Other/Do not wish to answer), ethnic background (Black or African descent, Hispanic or Latin American, White or European descent, Asian, Other), occupation (Unemployed, Student, Employed), history of physical and/or sexual abuse [15, 46], annual salary, and subjective socioeconomic status (SSE; “Your socio-economic status is… 1-low to 7-high”). An attention check question where participants were instructed to select a specific answer was used. Participants who failed it were excluded. The sociodemographic data descriptive statistics are presented in Table 1.

**Table 1:** Socio-demographic characteristics

	*n*	%
**Sex**		
Female	44	39.6
Male	67	60.4
**Gender**		
Woman	45	40.5
Man	66	59.5
**Occupation**		
Employed	98	88.3
Unemployed	8	7.2
Student	1	0.9
Other	4	3.6
**Ethnicity** ^**1**^		
White or European descent	93	83.8
Black or African descent	10	9.0
Hispanic or Latin American	3	2.7
Asian	2	1.8
Other	3	2.7
**Abuse History** ^**1**^ Sexual abuse	5	4.5
Physical abuse	9	8.1
No abuse reported	99	89.2
**Annual salary (USD)**		
0 to $20,000	14	12.6
$20,001 to $40,000	26	23.4
$40,001 to $60,000	32	28.8
$60,001 to $80,000	24	21.6
$80,001 to $100,000	12	10.8
More than $100,000	3	2.7
**Subjective socio-economic status**		
1	5	4.5
2	7	6.3
3	24	21.6
4	35	31.5
5	23	20.7
6	12	10.8
7	5	4.5

Note. ^1^Respondents could select more than one option for these questions

##### BP symptom severity

The Borderline Symptom List-23 [3] is a self-report instrument with good psychometric properties designed to assess borderline personality BP symptom severity through 23 items (e.g., “Criticism had a devastating effect on me”) rated on a 5-point Likert scale ranging from 0 (“not at all”) to 4 (“very strong”). BP symptom severity was measured as the average score on the BSL items, with higher values indicating more severe symptoms (Cronbach’s alpha and McDonald’s omega = 0.98).

##### Dispositional mentalization and affective empathy

The ACME is a 36-item measure with good psychometric properties evaluating dispositional mentalization (cognitive empathy), affective resonance, and affective dissonance (Vachon et al., 2014). Each item is rated on a 5-point Likert scale ranging from “Disagree Strongly” (1) to “Agree Strongly” (5). Mentalization pertains to understanding others’ emotions; Affective Resonance involves congruent emotional responses (e.g., feeling sadness when witnessing someone else’s sorrow); and Affective Dissonance refers to incongruent emotional reactions (e.g., deriving pleasure from another’s discomfort). Subscale scores were calculated by summing item responses, with higher scores indicating greater empathy (Cronbach’s alpha for Mentalization = 0.86, Affective Resonance = 0.88; Affective Dissonance = 0.97; McDonald’s omega for Mentalization = 0.84, Affective Resonance = 0.88; Affective Dissonance = 0.97).

##### Behavioral mentalization

**Fig. 1:**
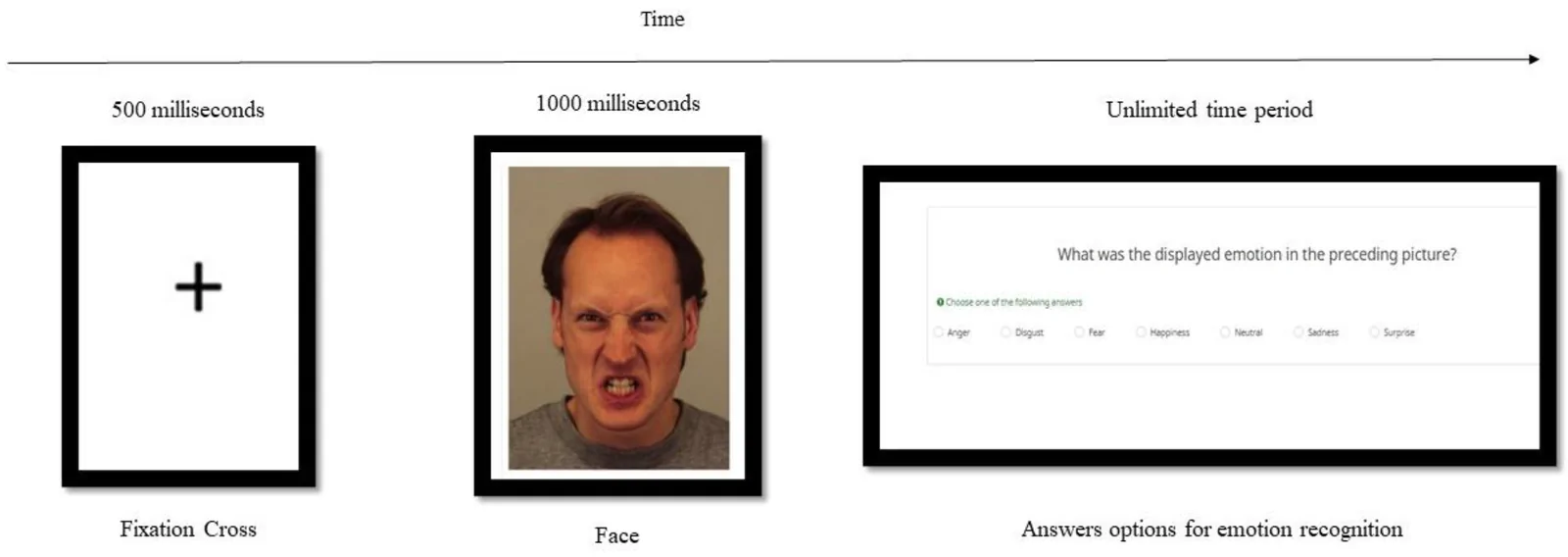
Time course of the emotion recognition task

An emotion recognition task was used to behaviorally assess mentalization abilities or skills. Overall, this methodology aligns with common practices in emotion recognition and social decision-making research (Mussel et al., 2014; [45]). Stimuli were 10 White adult faces (5 men and 5 women) selected from the Karolinska Directed Emotional Faces (KDEF) database (Lundqvist et al., 1998). The task comprised of 30 trials: each model featuring facial expressions of the three emotions that would be used in the Ultimatum Game: happiness, anger and neutrality. Each trial presented a fixation cross for 500 milliseconds, followed by a facial expression for 1000 milliseconds (112.4 × 152.4 pixels). Participants then identified the displayed emotion from six options: anger, neutrality, happiness, surprise, sadness or disgust. The additional response options were included to reduce guessing. Prior to the task, participants completed three practice trials with different faces representing each expression without feedback. Behavioral mentalization was measured with (a) the percentage of correct responses for each emotion, and (b) the misinterpretation patterns for each emotion using a confusion matrix. Figure 1 illustrates a trial sequence.

##### Social decision-making

**Fig. 2:**
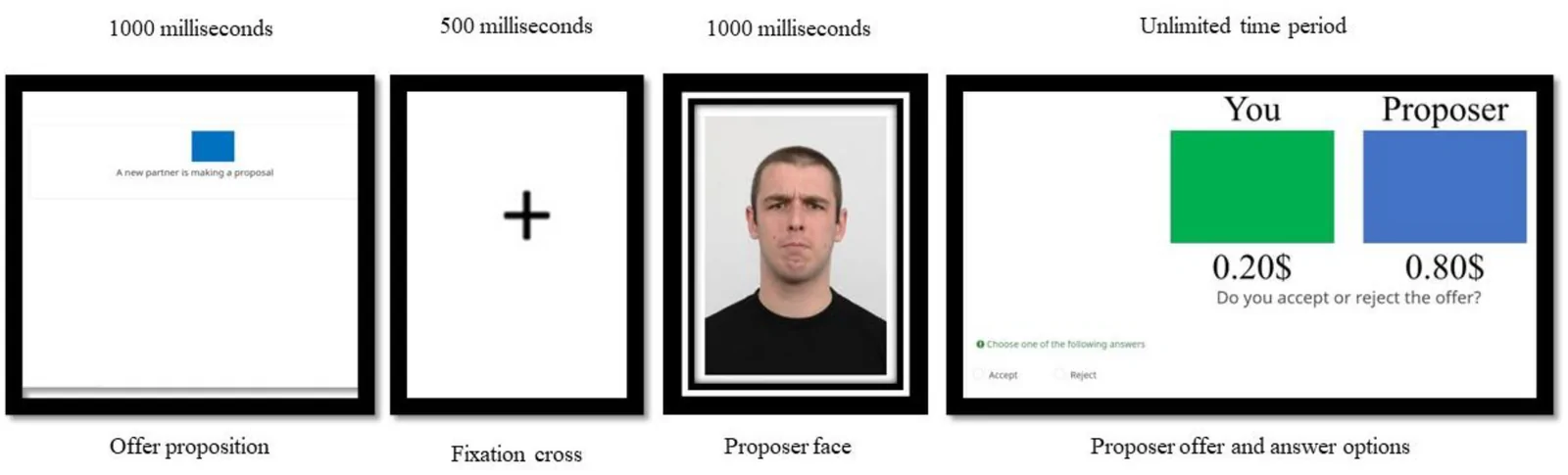
Time course of the UG task. Note. The last image showing the offers is static and presented with the choice to accept or reject. There was no time limit for selecting an answer option

Drawing on Polgár and colleagues [31], participants assumed the role of the Receiver in the Ultimatum Game (see Fig. 2), engaging with different Proposers whose facial expressions were displayed before their monetary offers of how to divide 1$ between themselves and the Receiver. Each UG trial began with a message stating “A new partner is making a proposal,” displayed for 1000 milliseconds, followed by a 500-millisecond fixation cross. Subsequently, the Proposer’s face appeared for 1000 milliseconds before the offer was presented. Offers were shown with colored squares indicating allocations: a green square labeled “You” for the Receiver’s share and a blue square labeled “Proposer” for the Proposer’s share. Participants chose to “Accept” or “Reject” the offer without any time constraints. Acceptance resulted in both parties receiving the proposed amounts, while rejection led to neither party receiving any money. Participants completed 66 trials, each involving a unique Proposer, with facial expressions categorized into three emotion conditions (22 trials per condition): neutrality, anger, or happiness. Each emotion was equally represented across male and female faces (11 trials each).

Unbeknownst to participants, the monetary offers were predetermined and divided into fair (“$0.50–$0.50” or “$0.60–$0.40”) and unfair (“$0.70–$0.30,” “$0.80–$0.20,” or “$0.90–$0.10”) categories. Fairness categories were defined based on previous studies (e.g., [10]) and on the fact that acceptance rates for offers around 40% to 50% of the endowment are very similar (e.g., Gabay et al., [14] but differ for lower percentages (typically 0 to 30%). Each emotional expression condition included four fair offer trials (two per offer type) and 18 unfair offer trials (six per offer type). This distribution aligns with prior research using the UG (e.g., O’Callaghan et al., [28]. Offers were randomly paired with Proposer faces from an image bank of 84 emotional expressions sourced from the Karolinska Directed Emotional Faces (KDEF) and Radboud Faces Database (RadF; [20]), distinct from those used in the emotion recognition task^2^. The random selection ensured varied distributions of images across participants. Trial order was randomized to reduce the probability of sequence effects.

To maintain consistent consideration of each trial and limit decisions based on cumulative earnings, participants were informed that one trial’s earnings (ranging from $0 to $1) would be randomly selected and added to their base payment of $4. Actually, all participants received an additional $1.

To favor attention to Proposer faces throughout the task, participants were told that five trials would include a follow-up question asking them to identify the Proposer’s displayed emotion, using the same response options as in the emotion recognition task. Before commencing the UG, participants completed a practice trial involving a Proposer’s offer and subsequent follow-up question about emotion identification. We measured the percentage of accepted offers for each displayed emotion (neutrality, anger, or happiness) separately for fair and unfair offers.

#### Analyses

##### BP symptom severity, mentalization and affective empathy

Hierarchical regressions were conducted to examine whether BP symptom severity statistically predicted mentalization and each facet of affective empathy (H1a-c). Potential covariates (age, sex, SSE) of mentalization and affective empathy measures were identified using preliminary correlational analyses. Covariates were included in the hierarchical regression models if they did not have high risk of multicollinearity with the BSL-23 score, i.e., a *r* ≥ .70 [42].

##### BP symptom severity and social decision-making

In order to test if BP symptom severity had an influence on decisions made during the UG, we ran a multilevel logistic regression using the glmer function in R. Specifically, our dependent variable was the participants’ choices (reject = 0, accept = 1) on each trial, our fixed factors were the participants’ BP symptom severity (centered BSL score), the Emotion of the Proposer (anger, neutrality and happiness; sum coded) and the Fairness of the offer (effect coded: fair = 0.5, unfair = -0.5) and their interactions. Our model also included a random intercept for participants and potential covariates (centered if continuous).

Preliminary analysis focused on the main effects of Emotion and Fairness to verify, as in Polgár and colleagues [31], that the probability of accepting fair offers was larger than accepting unfair offers, and that the probability of accepting offers from happy Proposers was higher than the probability of accepting those from neutral or angry ones. Direct contrasts between happiness vs. angry, happiness vs. neutrality and anger vs. neutrality using marginal means were used for the Emotion factor.

Our main hypotheses were tested by looking at the BSL symptom severity X Fairness interaction term (H2a) and BSL symptom severity X Emotion X Fairness interactions terms (H2b).

##### Mediating role of mentalization and affective empathy in the relation between bp symptom severity and social decision-making

Prior to testing mediation models, we ran preliminary analyses to examine whether candidate mediators were associated with sensitivity to emotional expressions in the Ultimatum Game (UG). Trials were nested within participants, and acceptance decisions were modeled as a binary outcome using multilevel logistic regression with a random intercept for participant. Sensitivity to emotional expressions was operationalized as the effect of proposer emotion on acceptance decisions. For each mediator (dispositional mentalization, behavioral mentalization as recognition of Happy Faces, behavioral mentalization as recognition of Angry Faces, affective resonance, and affective dissonance), two nested multilevel logistic regression models were estimated. The baseline model included main effects of Emotion, Fairness, the mediator, the interaction between Fairness and the mediator, and potential covariates. The comparison model additionally included the interaction between Emotion and the mediator. The contribution of the Emotion X mediator interaction was evaluated using likelihood ratio tests comparing the nested models. This approach allowed us to determine whether individual differences in mentalization or affective empathy were associated with differential sensitivity to proposer emotional expressions while accounting for the influence of fairness on decision-making.

Our hypothesis (H3a, b, c) were investigated with mediation analyses to test whether alterations in mentalization and affective empathy mediated the relationship between BP symptom severity and sensitivity to emotional expressions during the UG. Mediation was evaluated using a multilevel framework consistent with a product-of-coefficients approach (a × b) for each mediator separately. Path a represented the association between BP symptom severity and the mediator at the participant level. Path b represented the association between the mediator and sensitivity to emotional expressions in the multilevel logistic regression models. Indirect effects were estimated using Monte Carlo simulation to obtain confidence intervals.

Mediation analyses were conducted only if two conditions were met: (a) BP symptom severity was significantly associated with the mediator, and (b) the mediator significantly predicted sensitivity to emotional expressions in the multilevel models. If any of these conditions was not satisfied, mediation analyses were not performed.

## Results

### Descriptive statistics

Descriptive statistics for our main variables are found in Table 2. Regarding BP symptom severity, based on the classification by Kleindienst and colleagues [18], the mean BSL-23 score (1.07) in our sample corresponds to mild severity (with clinical patients usually associated with BSL-23 scores above 1.9).

**Table 2:** Descriptive statistics

	Min	Max	M	SD
Age^1^ (years)	22.00	72.00	39.40	11.67
BSL-23 Score	0.00	4.00	1.07	1.09
Dispositional Mentalization	18.00	60.00	44.17	7.92
Dispositional Resonance	28.00	60.00	46.19	8.97
Dispositional Dissonance	12.00	60.00	46.64	13.74
Percentage of happy faces identified correctly	0.00	100.00	87.74^2^	21.09
Percentage of angry faces identified correctly	0.00	100.00	60.36	26.25
Percentage of neutral faces identified correctly	10.00	100.00	64.41	19.20
Percentage of accepted offers	9.09	100.00	65.42	29.89
Percentage of fair offers accepted with a happy Proposer	0.00	100.00	95.27^2^	15.27
Percentage of unfair offers accepted with a happy Proposer	0.00	100.00	61.76	36.37
Percentage of Fair Offers Accepted with an Angry Proposer	0.00	100.00	91.44^2^	20.64
Percentage of Unfair Offers Accepted with an Angry Proposer	0.00	100.00	56.76	35.94
Percentage of fair offers accepted with a neutral Proposer	25.00	100.00	93.24^2^	17.82
Percentage of unfair offers accepted with a neutral Proposer	0.00	100.00	59.15	36.95
Difference between percentage of accepted fair offers from Happy vs. Angry Proposers	-25.00	75.00	3.83^2^	15.52
Difference between percentage of accepted unfair offers from Happy vs. Angry Proposers	-22.22	61.11	5.00^2^	13.54

Note. ^1^Some participants did not indicate their age so while *N* = 111 for all other variables, *n* = 107 for age. ^2^Indicates that the variable was not normally distributed. Nonparametric tests (e.g., Spearman correlations) were used with these variables

### Preliminary analyses to identify potential covariates

We examined correlations between participant characteristics (age, sex, SSE), the mentalization, affective empathy and Ultimatum Game (UG) variables (see Supplementary Table 1). In summary, older age was associated with higher affective resonance and affective dissonance scores, as well as higher dispositional mentalization. It was also linked to improved behavioral mentalization, specifically, more accurate recognition of happy facial expressions. In the UG, age was positively correlated with the acceptance rate of fair offers from happy Proposers. Female sex was positively associated with affective resonance and more accurate recognition of angry faces. Higher SSE was associated with lower affective resonance and affective dissonance, and poorer performance in behavioral mentalization: specifically, lower accuracy in identifying happy faces. In the UG, higher SSE was associated with reduced acceptance of fair offers from happy responders, but increased acceptance of unfair offers from happy responders and fair offers from neutral responders. Conversely, higher SSE was associated with decreased acceptance of fair offers from angry responders. These preliminary analyses lead us to consider age, sex assigned at birth and SSE in each analysis focusing on the variables they were found to be statistically correlated with.

### BP symptom severity relations with dispositional mentalization and affective empathy

Three hierarchical regressions were conducted to examine whether BP symptom severity statistically predicted mentalization and affective empathy facets beyond relevant covariates (Table 3).

For affective resonance, Step 1 with age, sex assigned at birth, and SSE explained 22.4% of the variance (*F*[3, 103] = 9.93, *p* < .001). Adding BP symptom severity in Step 2 significantly improved the model (Δ*R²* = 0.15), yielding a final *R²* = 0.37 (*F*[4, 102] = 15.31, *p* < .001). BP symptom severity negatively predicted affective resonance (*β* = –0.42, *p* < .001), with SSE not significant (*β* = − 0.16, *p* = .05).

For affective dissonance, Step 1 with age and SSE explained 21.7% of the variance (*F*[2, 104] = 14.39, *p* < .001). Adding BP symptom severity in Step 2 substantially improved the model (Δ*R²* = 0.42; *F*[3, 103] = 60.28, *p* < .001). The final model explained 63.7% of the variance in affective dissonance. BP symptom severity was a strong negative predictor (*β* = –0.69, *p* < .001) of affective dissonance. In the final model, SSE was also significant (*β* = –0.23, *p* = .001) but not age (*β* = 0.07, *p* = .284).

For mentalization, age alone in Step 1 predicted 7.8% of the variance, *F*(1, 105) = 8.88, *p* = .004. Adding BP symptom severity in Step 2 improved the model, Δ*R²* = 0.06, final *R²* = 0.13, *F*(2, 104) = 8.01, *p* < .001. BP symptom severity was a significant negative predictor (*β* = –0.24, *p* = .011), and age remained significant (*β* = 0.21, *p* = .026).

**Table 3:** Hierarchical Regressions for BP Symptom Severity Predicting Dispositional Mentalization and Affective Empathy

	B	95% CI B		S.E.	β	t	R^2^	DR^2^
		LL	UL					
**A. Affective resonance**								
Step 1							0.22	.22***
Constant	42.7	35.17	50.23	3.8		11.25		
Age	0.229	0.96	0.362	0.07	.03***	3.4		
Sex	3.78	0.641	6.921	1.58	-.21*	2.39		
SSE	-1.64	-2.76	-0.53	0.56	-.26**	-2.93		
Step 2							0.37	.15***
Constant	47.14	40.12	54.16	3.54		13.32		
Age	0.16	0.36	0.28	0.06	.21**	2.56		
Sex	2.39	-0.5	5.27	1.46	0.13	1.64		
SSE	-1.03	-2.06	0	0.52	-0.16	-1.98		
BSL-23 Score	-3.61	-5.05	-2.17	0.73	-.42***	-4.96		
**B. Affective dissonance**								
Step 1							0.22	.22***
Constant	51.5	40.41	62.58	5.59		9.21		
Age	0.26	0.06	0.45	0.1	.23**	2.62		
SSE	-3.48	-5.11	-1.85	0.82	-.37***	-4.24		
Step 2							0.63	.42***
Constant	62.02	54.21	69.84	3.94		15.73		
Age	0.08	-0.06	0.21	0.07	0.07	1.08		
SSE	-2.15	-3.29	-1.01	0.57	-.23***	-3.74		
BSL-23 Score	-8.7	-10.28	-7.13	0.8	-.69***	-10.92		
**C. Dispositional mentalization**								
Step 1							0.08	.08**
Constant	36.83	31.63	42.03	2.62		14.05		
Age	0.19	0.06	0.32	0.06	.28**	2.98		
Step 2							0.13	.06*
Constant	40.47	34.68	46.25	2.92		13.87		
Age	0.15	0.02	0.27	0.07	.21*	2.26		
BSL-23 Score	-1.88	-3.32	-0.43	0.73	-.24*	-2.58		

Note. S.E. = standard error of the estimate, β = Standardized coefficient, B = nonstandardized coefficient, CI = confidence intervals, LL = lower limit, UL = upper limit.*p < .05, **p< .01, ***p < .001

### BP symptom severity relations with behavioral mentalization

To examine whether BP symptom severity statistically predicts behavioral mentalization (i.e., emotion recognition abilities) beyond relevant covariates, three hierarchical regressions were conducted separately for each emotional expression (Table 4). For recognition of happy faces, Step 1with age and SSE explained 12.3% of the variance in the percentage of accurately identified happy faces (*F*[2, 104] = 7.28, *p* = .001). Including BP symptom severity significantly improved the model (Δ*R²* = 0.10). The final model explained 22.5% of the variance (*F*[3, 103] = 9.95, *p* < .001). BP symptom severity was a significant negative predictor of performance (*β* = –0.34, *p* < .001) as was SSE (*β* = –0.20, *p* = .028). For recognition of angry faces, Step 1 with sex assigned at birth explained 3.7% of the variance in the percentage of accurately identified angry faces (*F*[1, 109] = 4.23, *p* = .042). The addition of BP symptom severity led to a significant increase in explained variance (Δ*R²* = 0.05). The full model explained 7.5% of the variance in angry face recognition (*F*[2, 108] = 5.44, *p* = .006). BP symptom severity was a significant negative predictor (*β* = –0.24, *p* = .013). For recognition of neutral faces, the model including only BP symptom severity was not significant (*F*[1, 109] = 2.27, *p* = .135).

**Table 4:** Hierarchical regressions for BP symptom severity predicting behavioral mentalization

	B	95% CI B		S.E.	β	t	R^2^	DR^2^
		LL	UL					
**A. Recognition of happy faces**								
Step 1							0.12	.12**
Constant	91.47	72.84	110.09	9.39		9.74		
Age	0.33	0	0.66	0.17	.19*	2		
SSE	-4.05	-6.75	-1.28	1.38	-.27**	-2.91		
Step 2							0.23	.10***
Constant	99.7	81.55	117.85	9.15		10.9		
Age	0.19	-0.13	0.51	0.16	0.11	1.17		
SSE	-2.98	-5.62	-0.33	1.33	-.20*	-2.23		
BSL-23 Score	-6.81	-10.47	-3.14	1.85	-.34***	-3.68		
**B. Recognition of angry faces**								
Step 1							0.04	.04*
Constant	56.27	50	62.53	3.16		9.74		
Sex	10.32	0.37	20.27	5.02	.19*	2.06		
Step 2							0.08	.05*
Constant	63.23	55.05	71.42	4.13		15.32		
Sex	8.11	-1.75	17.98	4.98	0.15	1.63		
BSL-23 Score	-5.67	-10.1	-1.24	2.34	-.24*	-2.54		

Note. S.E. = standard error of the estimate, β = Standardized coefficient, B = nonstandardized coefficient, CI = confidence intervals, LL = lower limit, UL = upper limit.*p < .05, ** *p* < .01, ***p < .001

To extend the main analysis regarding behavioral mentalization, we examined overall error patterns in emotion recognition using a confusion matrix. This allowed us to identify which emotions were most frequently misclassified and to describe common misinterpretations beyond overall accuracy (Table 5). To explore whether BP symptom severity was linked to specific patterns of emotion misclassification, we examined hierarchical regressions between BSL-23 scores and key confusion types identified in the emotion recognition task above and beyond potential covariates (Supplementary Table 2).

**Table 5:**
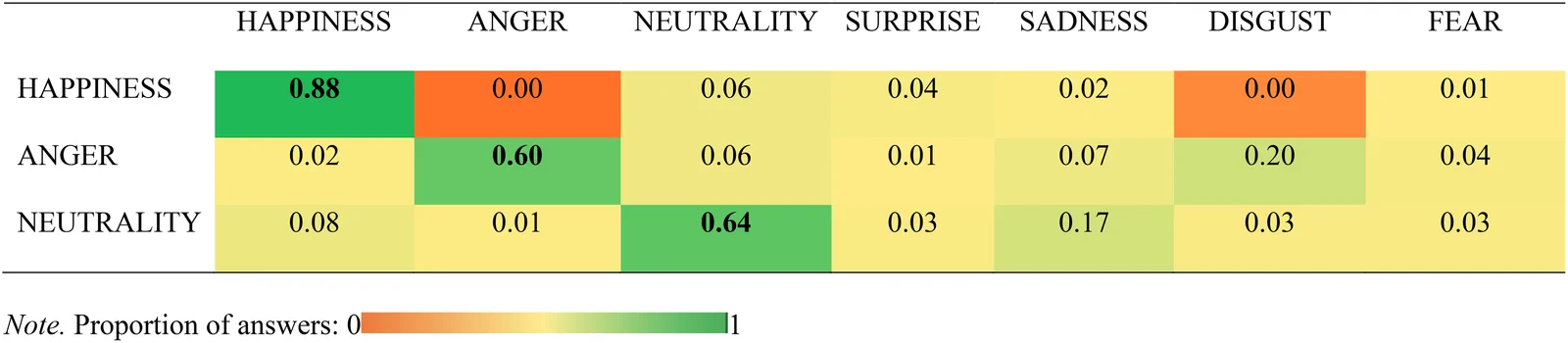
Confusion matrix for patterns of error in the emotion recognition task

Happiness expressions were the most accurately identified (88%), with few confusions—primarily with neutrality (6%) and surprise (4%). Anger expressions showed the lowest accuracy (60%), most often confused with disgust (20%), sadness (7%), and neutrality (6%), suggesting overlap mostly with other negative emotions. Neutral expressions accuracy was 64% and characterized by mistakenly identifying sadness (17%) and happiness (8%).

Overall, exploratory hierarchical regressions showed that BP symptom severity was a significant positive predictor of misclassifying happy faces as neutral (*β* = 0.27, *p* = .005) and as surprised (*β* = 0.26, *p* = .006) and misclassifying angry faces as sad (*β* = 0.33, *p* < .001). All results can be found in the supplementary material.

### BP symptom severity relations with social decision-making

#### Preliminary analysis

Looking at the Fairness main effect from our multilevel logistic regression model (including SSE and AGE as covariates; see Table 6), participants were substantially more likely to accept fair than unfair offers. The odds of acceptance were significantly higher for fair compared to unfair offers (odds ratio = 79.8, 95% CI [52.61, 121.05], *p* < .001; see Supplementary Fig. 1). The predicted probability of accepting fair offers was 0.99, compared to 0.72 for unfair offers.

Looking at the Emotion main effect, participants were more likely to accept offers from happy compared to angry proposers. The odds of acceptance were significantly higher for offers from happy compared to angry Proposers (odds ratio = 1.93, 95% CI [1.35, 2.77], *p* = .001; see Supplementary Fig. 1). The predicted probability of accepting offers from happy proposers was 0.97, compared to 0.94 for angry proposers. The odds of accepting offers from happy vs. neutral Proposers (odds ratio = 1.29, 95% CI [0.82, 2.04], *p* = .374) or from angry vs. neutral Proposers were not significantly different (odds ratio = 0.67, 95% CI [0.44, 1.01], *p* = .058). There was no Fairness X Emotion interaction.

#### Main analysis

Looking at the BP symptom severity X Fairness interaction (see Table 6), the higher the BSL score the more probability participant had of accepting offers and this effect was stronger for unfair offers.

There was no significant BP symptom severity X emotion or BP symptom severity X Emotion X Fairness interactions suggesting that, contrary to our hypothesis, BP symptom severity did not influence the sensitivity to the Proposers’ emotion during decision-making in the UG.

A significant interaction emerged between BP symptom severity and fairness, indicating that the relationship between BP symptom severity and acceptance decisions differed depending on whether offers were fair or unfair (odds ratio = 0.11, CI [0.08, 0.16], *p* < .001). Follow-up analyses (see Supplementary Fig. 2) examining the simple slopes of BSL within each fairness condition showed that BSL was not significantly associated with acceptance of unfair offers (odds ratio = 1.60, SE = 0.33, z = 1.42, CI [0.84, 3.04], *p* = .156). In contrast, higher BSL scores were associated with significantly lower odds of accepting fair offers (odds ratio = 0.18, SE = 0.36, z = − 4.81, CI [0.09, 0.36], *p* < .001). There was no significant BP symptom severity X emotion or BP symptom severity X Emotion X Fairness interactions suggesting that, contrary to our hypothesis, BP symptom severity did not influence the sensitivity to the Proposers’ emotion during decision-making in the UG.

**Table 6:** Results from the multilevel logistic regression on accept/reject choices in the Ultimatum Game

Predictor	β	SE	z	*p*	OR	lower CI	upper CI
*(Intercept)	*3.15*	*0.33*	*9.53*	*< 0.001*	*23.35*	*12.21*	*44.62*
BP symptom severity (centered)	*-0.63*	*0.33*	*-1.88*	*0.06*	*0.53*	*0.28*	*1.03*
*Emotion 1	*-0.34*	*0.10*	*-3.51*	*< 0.001*	*0.71*	*0.58*	*0.86*
*Emotion 2	*0.30*	*0.11*	*2.83*	*0.005*	*1.36*	*1.10*	*1.68*
*Fairness (effect coded)	*4.38*	*0.21*	*20.60*	*< 0.001*	*79.80*	*52.61*	*121.05*
SSE (centered)	*0.24*	*0.23*	*1.02*	*0.31*	*1.27*	*0.80*	*2.01*
Age (centered)	*-0.00*	*0.03*	*-0.04*	*0.97*	*1.00*	*0.95*	*1.06*
BP symptom severity X Emotion 1	*0.13*	*0.09*	*1.47*	*0.14*	*1.13*	*0.96*	*1.34*
BP symptom severity X Emotion 2	*-0.00*	*0.09*	*-0.04*	*0.97*	*0.10*	*0.83*	*1.191*
*BP symptom severity X Fairness	*-2.19*	*0.17*	*-12.93*	*< 0.001*	*0.11*	*0.08*	*0.16*
Emotion 1 X Fairness	*-0.29*	*0.20*	*-1.49*	*0.14*	*0.75*	*0.51*	*1.10*
Emotion 2 X Fairness	*0.19*	*0.22*	*0.90*	*0.37*	*1.21*	*0.79*	*1.85*
BP symptom severity X Emotion 1 X Fairness	*0.20*	*0.17*	*1.17*	*0.24*	*1.22*	*0.88*	*1.70*
BP symptom severity X Emotion 2 X Fairness	*0.08*	*0.18*	*0.42*	*0.68*	*1.08*	*0.76*	*1.54*

Note. BP: Borderline Personality (measured with the BSL-23); SSE: Subjective socioeconomic status; OR: Odds ratio; CI: 95% confidence intervals for OR calculated with Wald CI. Significant factors (*p* < .05) are indicated by *.

### Mediating role of mentalization and affective empathy in the relationship between BP symptom severity and social decision-making

#### Preliminary analysis

To determine whether candidate mediators were associated with sensitivity to emotional expressions in the Ultimatum Game, we compared nested multilevel logistic regression models predicting acceptance decisions. The baseline models included main effects of proposer emotion, fairness, the mediator, and the fairness-by-mediator interaction, along with covariates and a random intercept for participant. Comparison models additionally included the emotion-by-mediator interaction. Likelihood ratio tests indicated that the inclusion of the emotion-by-mediator interaction did not significantly improve model fit for any mediator indicating that dispositional mentalization (*X*^*2*^(2) = 1.40, *p* = .496), behavioral mentalization (recognition of Happy Faces: *X*^*2*^(2) = 0.66, *p* = .719; recognition of Angry Faces: *X*^*2*^(2) = 0.07, *p* = .966), affective resonance (*X*^*2*^(2) = 0.27, *p* = .875), and affective dissonance (*X*^*2*^(2) = 1.33, *p* = .512) were not associated with differential sensitivity to proposer emotional expressions in acceptance decisions.

#### Main analysis

Consistent with our analytic plan, mediation analyses were conducted only when (a) borderline personality symptom severity was significantly associated with the mediator and (b) the mediator significantly predicted sensitivity to emotional expressions. Because the second criterion was not met for any mediator, mediation analyses were not conducted.

## Discussion

This study aimed to investigate the relationship between BP symptom severity, mentalization and affective empathy (both dispositional and behavioral) and social decision-making using the Ultimatum Game (UG) in a community sample. Specifically, we explored whether BP symptom severity influenced mentalization and affective empathy and decision-making behaviors when interacting with Proposers expressing different emotions (happiness, anger, neutrality) in an UG. We also wanted to explore the mediating roles of mentalization and affective empathy in the relationship between BP symptom severity and social decision-making in the UG. Our findings offer valuable insights into these relationships, though only some of our hypotheses were supported.

### BP symptom severity, mentalization and affective empathy

As hypothesized, higher BP symptom severity was significantly associated with lower levels of dispositional mentalization and affective empathy (affective resonance, and affective dissonance). These results align with previous research suggesting that BP disorder is characterized by impairments in both dispositional mentalization and affective components of empathy [36]. However, especially for affective empathy, Salgado and colleagues’ [36] systematic review also highlights that findings in the field using the Interpersonal Reactivity Index (IRI; [9]) greatly vary: with some studies finding impairments (e.g., Homan et al., [17], no effects (e.g., Petersen et al., [30] or even enhanced empathy (e.g., Guttman and Laporte, [16]; see also the meta-analysis of [2]) in BP disorder. Here, we used the ACME (Vachon et al., 2016), a relatively new validated tool to assess self-reported dispositional empathy and tested if BP symptom severity in a community sample explained variance in dispositional affective empathy above and beyond other potential covariates (age, sex assigned at birth, and subjective social economic status). A previous study in a community sample using the ACME and the BSL-23 also found a relation between BP symptom severity and lower affective dissonance scores indicating an increased tendency to experience emotions that are incongruent with those of others [23]. These convergent findings suggest that impairments associated to BP symptom severity may be characterized by incongruent emotional reactions (e.g., deriving pleasure from another’s discomfort) and that affective dissonance could be a promising avenue in the study of affective empathy in BP disorder. However, Maheux-Caron and colleagues [23] did not find a significant relation with dispositional affective resonance nor dispositional mentalization. The lack of consistent results using the ACME resonance scale and the important number of studies finding no difference when using other self-reported affective empathy measures [36] suggests that this dimension of empathy may generally be unaffected by BP symptom severity. Although our results about dispositional mentalization are inconsistent with Maheux-Caron and colleagues’ [23], they align with Salgado and colleagues’ [36] findings that out of 10 studies that used the Perspective-taking subscale of the IRI, seven reported impairments in BP patients compared to healthy controls. Our results also add to this literature by showing that mentalization seems to be negatively associated with BP symptom severity even at the trait level in a community sample.

Our behavioral mentalization measure using an emotion recognition task reinforced these findings, suggesting that higher BP symptom severity was associated with poorer performance in recognizing happiness and anger. This is at odds with Bora’s meta-analysis (2024) that did not find impairments in emotion recognition in BP disorder. Importantly, while we used images presenting the entire face, results from Bora (2024) used the Reading the mind in the eye test which only present the eye region. Our results are however in line with prior findings from another meta-analysis that included studies where full faces were presented [8] showing that patients diagnosed with BP disorder were less accurate than controls in recognizing anger (but see [24]), we also observed a significant negative correlation between BP symptom severity and the ability to recognize angry faces. Notably, our results extend these findings by showing that BP symptom severity is also associated with difficulties in recognizing facial expression of anger at the trait level in a community sample above and beyond the influence of other covariables such as sex assigned at birth. By characterizing the pattern of misclassifications, we were also able to show that BP symptom severity was associated with a tendency to identify anger as sadness and as neutral facial expressions.

Contrary to Daros and colleagues’ [8] findings, we found that BP symptom severity was also associated with poorer performance in recognizing happy facial expressions (specifically by misidentifying it as surprise and neutral facial expressions). Furthermore, while the most important deficit found in their meta-analysis was for neutral facial expressions, we did not find a significant association between BP symptom severity and recognition of neutral expressions. These divergences may stem from methodological differences: while their meta-analysis focused on individuals with a formal BP diagnosis, our study assessed BP symptom severity in a nonclinical or mixed sample. Additionally, we used continuous symptom severity scores rather than categorical group comparisons, which may affect sensitivity to specific emotion recognition impairments. Finally, the facial expression stimuli in the studies they considered were different from ours (e.g., Pictures of Facial Affect; [11]). Since our dataset is relatively small compared to theirs, our results should be considered carefully and replicated.

Overall, alterations in mentalization and affective empathy reported here could partially explain interpersonal misunderstandings commonly reported in BP disorder [4, 21], which could be also found to a lesser extent at the subclinical trait level.

### Association of BP symptom severity with social decision-making in the UG

Consistent with a study using a very similar task [31], our participants overall accepted fair offers more frequently than unfair offers, and emotional expressions of Proposers significantly influenced acceptance decisions—offers from happy Proposers were accepted more often compared to offers from angry Proposers, whereas acceptance rates did not significantly differ between happy and neutral Proposers or between angry and neutral Proposers. Our results are in line with established findings that positive emotional cues facilitate cooperative behaviors [26, 31] and adds support to theories proposing that emotional facial expressions carry communicative information that is processed to adjust decision-making during social interactions (e.g., Frith, [13].

Our results also suggest that BP symptom severity interacted significantly with the fairness of the offer. Follow-up analyses showed that BP symptom severity was not significantly associated with acceptance of unfair offers, but higher BP symptom severity was associated with lower acceptance of fair offers. Thus, the relation between BP symptom severity and acceptance decisions was specific to fair offers and did not generalize across all offer types. This pattern is different from our original expectation. Based on prior results on individuals with a BP diagnostic [31], we expected greater borderline symptom severity to be linked to increased acceptance of unfair offers, possibly reflecting heightened rejection sensitivity (e.g., Staebler et al., [40], a tendency to avoid interpersonal conflict, or a reduced sense of deservingness (e.g., Kot et al., [19], Rüsch, [35]. However, the present multilevel analyses did not support that interpretation. Instead, individuals with higher borderline symptom severity appeared less likely to accept fair offers. The divergence between our results and the one from Polgár and colleagues [31] may be explained by differences in the studied samples. Importantly, Polgár and colleagues [31] tested differences between healthy controls and patients who, as a group, had much more severe BP symptoms than the community participants from our study. Indeed, our sample had a mean score of 1.07 on the BSL-23, which corresponds to a mild severity classification based on categories proposed by Kleindienst et al., [18], well below the 1.9 threshold for high severity. This suggests that effect of BP symptom severity on fairness-related decision-making may be dependent on the level of BP symptoms severity (mild vs. more severe or clinical levels). One possible interpretation for our results is that, in our community sample, borderline symptom severity may be associated less with tolerance of unfair treatment than with a broader alteration in how even objectively fair social exchanges are evaluated. Fair offers may not necessarily be experienced as straightforwardly positive or trustworthy by individuals with elevated borderline features. More generally, this finding may reflect a subtle disruption in social appraisal processes rather than a simple increase in willingness to accept disadvantageous treatment. Importantly, this interpretation should remain tentative. Because acceptance of fair offers was near ceiling overall, even a statistically reliable decrease in acceptance among participants with higher BSL scores occurred against a background of very high acceptance rates. Thus, the effect is best understood as a shift within a generally cooperative response pattern, rather than as evidence that individuals with elevated borderline symptoms routinely reject fair treatment. Still, the fact that borderline symptom severity was associated with decisions specifically in the fair condition suggests that symptom severity may shape how socially positive or non-threatening situations are processed. Our findings should also be interpreted cautiously in light of recent evidence suggesting limited and inconsistent effects of borderline pathology on Ultimatum Game behavior. A recent mini meta-analysis of four studies, including one using neutral images of proposers, did not find reliable differences in acceptance rates between individuals with borderline personality disorder and comparison groups [10]. Taken together, these findings suggest that the influence of borderline symptoms on fairness-related decision-making may be subtle, context-dependent, or contingent on symptom severity. Additional research, particularly using well-powered designs and samples spanning a broader range of symptom severity, is needed to clarify the conditions under which borderline symptomatology meaningfully affects social decision-making.

Contrary to our hypotheses, BP symptom severity did not moderate the effect of proposer emotion on decisions in the UG. Neither the BP symptom severity X Emotion interaction nor the three-way BP symptom severity X Emotion X Fairness interaction was significant. Thus, although proposer emotion influenced decisions overall, this emotional influence did not vary as a function of borderline symptom severity in our community sample. In other words, higher levels of borderline symptoms were not associated with greater or lesser sensitivity to the proposer’s emotional expression during decision-making. This finding differs from expectations based on Polgár et al., [31], who reported altered responses to emotional cues in patients with borderline personality disorder. Again, one likely explanation is the difference in sample characteristics—clinical vs. community samples. It is therefore possible that alterations in emotion-related social decision-making emerge more clearly only at higher, clinically more severe levels of borderline pathology.

Taken together, the present findings suggest that, in a community sample with predominantly mild borderline symptom severity, borderline features do not appear to influence emotional sensitivity during social decision-making in the UG. Instead, their role may be more closely tied to how fairness itself is processed, particularly in situations that would typically invite acceptance. This distinction is important, because it suggests that previously reported social decision-making alterations in borderline personality disorder may not reflect a generalized hypersensitivity to emotional expressions, but may depend on symptom severity, clinical status, and the specific interpersonal meaning of the social exchange.

### Mediating role of mentalization and affective empathy in the relation between BP symptom severity and social decision-making

As mentioned before, no significant relation was found between BP symptom severity and behavioral patterns related to adjustment to emotions or the Proposers in the UG. Furthermore, no relation was found between mentalization or affective empathy and behavioral adjustments in the UG preventing the potential (full) mediation. This contradicted our hypothesis that BP symptom severity’s influence on the effects of Proposers’ emotional facial expressions in the UG [31] would be mediated by alterations in mentalization or affective empathy. A potential explanation for this null result could be that alterations in mentalization and affective empathy and social decision-making in BP symptomatology are affected by independent underlying mechanisms.

### Limitations and future directions

Some limitations and constraints to the generalizability of our findings should be considered. This study was cross-sectional and online. Future research using experimental or longitudinal methods may help clarify the causal relationships between BP symptom severity, mentalization, affective empathy, and social decision-making. Although attention checks were implemented, conducting the study in a controlled laboratory setting could further reduce concerns related to online data collection (e.g., attentional lapses, misinterpretation of instructions, or automated responses). Additionally, the U.S.-based sample limits the cultural generalizability of the findings, and the use of volunteer sampling may introduce selection bias. Furthermore, the use of a community sample and the assessment of BP symptom severity rather than clinical diagnoses limits the generalizability of our findings to clinical populations. Second, given the exploratory nature of the analyses involving multiple comparisons, the identified patterns of emotional misclassification require replication before strong conclusions can be drawn. Third, we did not include a broader range of emotional expressions, such as disgust, which may be particularly relevant in individuals with BP disorder [19]. Fourth, our computerized task presenting still pictures of faces of strangers may not be engaging enough to trigger the responses usually associated BP symptomatology.

Future studies should include clinical populations and employ longitudinal designs and more ecological tasks (e.g., face to face interactions with real Proposers) to explore how mentalization or affective empathy deficits evolve alongside BP symptom severity and social decision-making over time. Additionally, employing neuroimaging techniques could illuminate underlying neural mechanisms driving observed mentalization, affective empathy and decision-making differences.

## Conclusion

BP symptom severity, assessed in a community sample, was robustly associated with mentalization and affective empathy deficits, particularly affecting mentalization—measured both at the dispositional and behavioral levels—and affective dissonance. In the Ultimatum Game, BP symptom severity was linked to a specific pattern of social decision-making: individuals with higher symptom severity were less likely to accept fair offers, while responses to unfair offers and emotional facial expressions were not significantly affected. These findings suggest that social decision-making differences associated with BP symptoms may reflect altered responses to fairness or expectations of reciprocity rather than a generalized deficit in emotion processing. These findings contribute to a nuanced understanding of social cognition in BP, highlighting mentalization and affective empathy impairments as important components of the symptomatology, even if their role in social decision-making seems more selective than previously assumed.

## Supplementary Information

Below is the link to the electronic supplementary material.

Supplementary Material 1

## Data Availability

The datasets used and/or analysed during the current study are available from the corresponding author on reasonable request.

## Footnotes

1 Note that mediation analyses were only run if (a) BP symptom severity was found to be significantly related to mentalization or affective empathy measures, and (b) if mentalization or affective empathy measures (mediators) were significantly related to social decision-making in the UG.

2 Specifically, for angry expressions, 16 faces were from KDEF and 12 from RadF; for happy expressions, 15 from KDEF and 13 from RadF; and for neutrality expressions, 17 from KDEF and 11 from RadF.

## Abbreviations

ACME: Affective and Cognitive Measure of Empathy
AMPD: Alternative Model for Personality Disorders
ANCOVA: Analysis of Covariance
ANOVA: Analysis of Variance
APA: American Psychiatric Association
BP: Borderline Personality
BPD: Borderline Personality Disorder
BSL: 23–Borderline Symptom List–23
CEREP: Comité d’éthique de la recherche en éducation et psychologie (Ethics Committee for Research in Education and Psychology, Université de Montréal)
CI: Confidence Interval
DSM: 5 / DSM–5–TR–Diagnostic and Statistical Manual of Mental Disorders, 5th Edition / Text Revision
IRI: Interpersonal Reactivity Index
KDEF: Karolinska Directed Emotional Faces
RadF: Radboud Faces Database
SD: Standard Deviation
S.E.: Standard Error
SSE: Subjective Socioeconomic Status
UG: Ultimatum Game
USD: United States Dollar
