# Supplementary material for: Borderline personality symptom severity predicts reduced mentalization and affective empathy and altered responses to fairness in the Ultimatum Game
Source: Borderline Personal Disord Emot Dysregul. 2026 Jul 8;13:31. doi: 10.1186/s40479-026-00358-y (PMC13628828; doi:10.1186/s40479-026-00358-y)
Supplement: Supplementary file 1 [file 40479_2026_358_MOESM1_ESM.docx]

**Supplementary Table 1**

*Correlation between Age, Sex, SSE and our main variables*

|  | 1 | 2 | 3 | 4 | 5 | 6 | 7 | 8 | 9 | 10 | 11 | 12 | 13 | 14 | 15 | 16 |
| --- | --- | --- | --- | --- | --- | --- | --- | --- | --- | --- | --- | --- | --- | --- | --- | --- |
| 1. Age | 1 | .088 | -.149 | .279** | .358** | .285** | .112 | .055 | -.145 | -.046 | .204* | -.076 | .12 | -.069 | .179 | -.05 |
| 2.Sex | .088 | 1 | .099 | .127 | .220* | .178 | .072 | .193* | .14 | -.025 | .048 | -.015 | -.019 | -.056 | .018 | -.015 |
| 3.SSE | -.149 | .099 | 1 | -.024 | -.290** | -.419** | -.239* | -.158 | -.157 | .209* | -.196* | .263** | -.260** | .189* | -.184 | .239* |
| 4.Dispositional Mentaliztion | .279** | .127 | -.024 | 1 | .593** | .390** | .261** | .119 | .046 | -.017 | .202* | -.069 | .251** | -.02 | .210* | -.028 |
| 5.Dispositional Resonance | .358** | .220* | -.290** | .593** | 1 | .760** | .263** | .294** | -.04 | -.129 | .169 | -.161 | .256** | -.143 | .232* | -.145 |
| 6.Dispositional Dissonance | .285** | .178 | -.419** | .390** | .760** | 1 | .414** | .344** | .006 | -.199* | .249** | -.244* | .344** | -.206* | .300** | -.229* |
| 7.Percentage of happy faces identified correctly | .112 | .072 | -.239* | .261** | .263** | .414** | 1 | .406** | .157 | -.196* | .106 | -.217* | .095 | -.185 | .139 | -.194* |
| 8.Percentage of angry faces identified correctly | .055 | .193* | -.158 | .119 | .294** | .344** | .406** | 1 | .105 | -.14 | -.061 | -.133 | -.06 | -.146 | .073 | -.135 |
| 9.Percentage of neutral faces identified correctly | -.145 | .14 | -.157 | .046 | -.04 | .006 | .157 | .105 | 1 | -.063 | -.018 | -.037 | -.171 | -.09 | .027 | -.05 |
| 10.Percentage of accepted offers | -.046 | -.025 | .209* | -.017 | -.129 | -.199* | -.196* | -.14 | -.063 | 1 | .240* | .969** | .194* | .981** | .214* | .987** |
| 11.Percentage of fair offers accepted with a happy Proposer | .204* | .048 | -.196* | .202* | .169 | .249** | .106 | -.061 | -.018 | .240* | 1 | .155 | .644** | .181 | .797** | .203* |
| 12.Percentage of unfair offers accepted with a happy Proposer | -.076 | -.015 | .263** | -.069 | -.161 | -.244* | -.217* | -.133 | -.037 | .969** | .155 | 1 | .097 | .930** | .14 | .957** |
| 13.Percentage of Fair Offers Accepted with an Angry Proposer | .12 | -.019 | -.260** | .251** | .256** | .344** | .095 | -.06 | -.171 | .194* | .644** | .097 | 1 | .181 | .601** | .12 |
| 14.Percentage of Unfair Offers Accepted with an Angry Proposer | -.069 | -.056 | .189* | -.02 | -.143 | -.206* | -.185 | -.146 | -.09 | .981** | .181 | .930** | .181 | 1 | .169 | .965** |
| 15.Percentage of fair offers accepted with a neutral Proposer | .179 | .018 | -.184 | .210* | .232* | .300** | .139 | .073 | .027 | .214* | .797** | .14 | .601** | .169 | 1 | .158 |
| 16.Percentage of unfair offers accepted with a neutral Proposer | -.05 | -.015 | .239* | -.028 | -.145 | -.229* | -.194* | -.135 | -.05 | .987** | .203* | .957** | .12 | .965** | .158 | 1 |

*Note.* For normally distributed variables, Pearson’s correlations were used, for others, Spearman’s. SSE: subjective socioeconomic status.

* *p* < .05; ** *p* < .01; ** *p* < .001

**Regression analyses looking at associations of BP Symptom Severity with misclassification patterns**

For misclassification of happy faces as neutral, Step 1 with age and SSE explained 13.6% of the variance (*F*[2, 104] = 8.16, *p* < .001). The addition of BP symptom severity led to a significant increase in explained variance (Δ*R²* = .07). The full model explained 17.7% of the variance (*F*[3, 103] = 8.61, *p* < .001). BP symptom severity was a significant positive predictor (*β* = .27, *p* = .005) as was SSE (*β* = .21, *p* = .023). For misclassification of happy faces as surprise, the model including only BP symptom severity explained 6.7% (*F*[1, 109] = 7.77, *p* = .006), with BP symptom severity as a significant positive predictor (*β* = .26, *p* = .006).

For misclassification of angry faces as disgusted, Step 1 with age explained 6.0% of the variance (*F*[1, 105] = 6.66, *p* = .011). The addition of BP symptom severity led to a significant increase in explained variance (Δ*R²* = .02). The full model explained 8.3% of the variance (*F*[2, 104] = 4.72, *p* = .011). While age was a positive predictor (*β* = .20, *p* = .041), BP symptom severity was not (*β* = -.16, *p* = .105). For misclassification of angry faces as sadness, Step 1 with SSE explained 5.0% of the variance (*F*[1, 109] = 6.84, *p* < .010). The addition of BP symptom severity led to a significant increase in explained variance (Δ*R²* = .10). The full model explained 16.0% of the variance (*F*[2, 108] = 10.27, *p* < .001). BP symptom severity was a significant positive predictor (*β* = .33, *p* < .001). For misclassification of angry faces as neutral, Step 1 with age explained 6.6% of the variance (*F*[1, 105] = 7.47, *p* = .007). The addition of BP symptom severity led to a significant increase in explained variance (Δ*R²* = .06). The full model explained 13.0% of the variance (*F*[2, 104] = 7.76, *p* < .001). BP symptom severity was a significant positive predictor (*β* = .26, *p* = .007).

For misclassification of neutral faces as sad, Step 1 with age explained 5.8% of the variance (*F*[1, 105] = 6.47, *p* = .012). The addition of BP symptom severity did not significantly increase explained variance (Δ*R²* = .00). For misclassification of neutral faces as happy, the model including BP symptom severity was not significant (*p* = .605).

Detailed results are presented in Supplementary Table 2

**Supplementary Table 2**

*Hierarchical Regressions for BP Symptom Severity Predicting Misclassifications in the Emotion Recognition Task*

A. Happiness Misclassified as Neutrality

|  | | B | 95% CI B | | S.E. | β | *t* | *R^2^* | *ΔR^2^* |
| --- | --- | --- | --- | --- | --- | --- | --- | --- | --- |
|  | |  | LL | UL |  |  |  |  |  |
|  | Step 1 |  |  |  |  |  |  | .14 | .14*** |
| 1 | Constant | 0.05 | -0.07 | 0.18 | 0.61 |  | 0.89 |  |  |
|  | Age | 0.00 | -0.01 | 0.00 | 0.00 | -.22* | -2.37 |  |  |
|  | SSE | 0.03 | 0.01 | 0.04 | 0.01 | .27** | 2.88 |  |  |
|  | Step 2 |  |  |  |  |  |  | .20 | .07** |
| 2 | Constant | 0.01 | 55.05 | -0.11 | 0.13 |  | 0.19 |  |  |
|  | Age | 0.00 | -1.75 | 0.00 | 0.00 | -.15 | -1.69 |  |  |
|  | SSE | 0.02 |  | 0.00 | 0.04 | .21* | 2.30 |  |  |
|  | BSL-23 Score | 0.04 | -10.10 | 0.01 | 0.06 | .27** | 2.89 |  |  |

B. Happiness Misclassified as Surprise

|  | | B | 95% CI B | | S.E. | β | *t* | *R^2^* | *ΔR^2^* |
| --- | --- | --- | --- | --- | --- | --- | --- | --- | --- |
|  | |  | LL | UL |  |  |  |  |  |
|  | Step 1 |  |  |  |  |  |  | .07 | .07** |
| 1 | Constant | 0.02 | -.00 | 0.04 | 0.01 |  | 1.61 |  |  |
|  | BSL-23 Score | 0.02 | 0.01 | 0.03 | 0.01 | .26** | 2.79 |  |  |

|  | | B | 95% CI B | | S.E. | β | *t* | *R^2^* | *ΔR^2^* |
| --- | --- | --- | --- | --- | --- | --- | --- | --- | --- |
|  | |  | LL | UL |  |  |  |  |  |
|  | Step 1 |  |  |  |  |  |  | .06 | .06* |
| 1 | Constant | 0.05 | -0.07 | 0.17 | 0.60 |  | 0.89 |  |  |
|  | Age | 0.00 | 0.00 | 0.01 | 0.00 | .24* | 2.58 |  |  |
|  | Step 2 |  |  |  |  |  |  | .20 | .07** |
| 2 | Constant | 0.11 | -0.03 | 0.24 | 0.07 |  | 1.57 |  |  |
|  | Age | 0.00 | 0.00 | 0.01 | 0.00 | .20* | 2.07 |  |  |
|  | BSL-23 Score | -0.03 | -0.06 | 0.01 | 0.02 | -.16 | -1.64 |  |  |

C. Anger Misclassified as Disgust

D. Anger Misclassified as Sadness

|  | | B | 95% CI B | | S.E. | β | *t* | *R^2^* | *ΔR^2^* |
| --- | --- | --- | --- | --- | --- | --- | --- | --- | --- |
|  | |  | LL | UL |  |  |  |  |  |
|  | Step 1 |  |  |  |  |  |  | .06 | .06* |
| 1 | Constant | -0.02 | -0.08 | 0.05 | 0.04 |  | 0.89 |  |  |
|  | SSE | 0.02 | 0.01 | 0.04 | 0.01 | .24* | 2.62 |  |  |
|  | Step 2 |  |  |  |  |  |  | .16 | .10*** |
| 2 | Constant | -0.02 | -0.09 | 0.04 | 0.03 |  | -0.70 |  |  |
|  | SSE | 0.01 | 0.00 | 0.03 | 0.01 | .15 | 1.68 |  |  |
|  | BSL-23 Score | 0.04 | 0.02 | 0.06 | 0.01 | .33*** | 3.60 |  |  |

E. Anger Misclassified as Neutrality

|  | |  | LL | UL |  |  |  |  |  |
| --- | --- | --- | --- | --- | --- | --- | --- | --- | --- |
|  | Step 1 |  |  |  |  |  |  | .07 | .07** |
| 1 | Constant | 0.18 | 0.09 | 0.27 | 0.05 |  | 3.96 |  |  |
|  | Age | 0.00 | -0.01 | 0.00 | 0.00 | -.26** | -2.73 |  |  |
|  | Step 2 |  |  |  |  |  |  | .13 | .06** |
| 2 | Constant | 0.11 | 0.01 | 0.21 | 0.05 |  | 2.25 |  |  |
|  | Age | 0.00 | 0.00 | 0.00 | 0.00 | -.19 | -1.98 |  |  |
|  | BSL-23 Score | 0.03 | 0.01 | 0.06 | 0.01 | -.26** | 2.75 |  |  |

*Note*. S.E. = standard error of the estimate, β = Standardized coefficient, B = nonstandardized coefficient, CI = confidence intervals, LL = lower limit, UL = upper limit.

**p* < .05, ** *p* < .01, ****p* < .001

**Supplementary Figure 1**

*Predicted probability of acceptance in the UG for fair and unfair offers from Proposers expressing different emotions*

*Note.* UG: ultimatum game. Error bars represent 95% confidence intervals.

**Supplementary Figure 2**

*Predicted probability of acceptance in the UG for fair and unfair offers for varying BP severity level*

*Note.* Shaded areas represent 95% confidence intervals estimated from the logistic regression model.
